# Altered regional brain white matter in dry eye patients: a brain imaging study

**DOI:** 10.18632/aging.203976

**Published:** 2022-03-24

**Authors:** Yun-Qing Luo, Rong-Bin Liang, San-Hua Xu, Yi-Cong Pan, Qiu-Yu Li, Hui-Ye Shu, Min Kang, Pin Yin, Li-Juan Zhang, Yi Shao

**Affiliations:** 1 Department of Ophthalmology, The Second Affiliated Hospital of Nanchang University, Jiangxi Province Ocular Disease Clinical Research Center, Nanchang 330006, Jiangxi, PR China; 2 Department of Ophthalmology, The First Affiliated Hospital of Nanchang University, Jiangxi Province Medical Imaging Research Institute, Nanchang 330006, Jiangxi, PR China

**Keywords:** dry eye, white matter, diffusion tensor imaging, neurite orientation dispersion, density imaging

## Abstract

This study aimed to investigate the regional changes of brain white matter (WM) in DE patients using diffusion tensor imaging (DTI) and neurite orientation dispersion and density imaging (NODDI). A total of 25 dry eye patients (PAT) and 25 healthy controls (HC) were recruited. All subjects underwent DTI and NODDI, fractional anisotropy (FA), mean diffusivity (MD), radial diffusivity (RD), isotropic volume fraction (FISO), intra-cellular volume fraction (FICVF), and orientation dispersion index (ODI) were obtained respectively. Then complete Hospital Anxiety and Depression Scale (HADS), anxiety score (AS) or depression scores (DS) were obtained. Receiver operating characteristic (ROC) curve analysis was used to evaluate the reliability of DTI and NODDI in distinguishing the two groups. DTI revealed that PAT had lower FA in both the left superior longitudinal fasciculus (LSLF) and the corpus callosum (CC), and higher MD in the LSLF, the right posterior limb of the internal capsule and the right posterior thalamic radiation. PAT had significant AD changes in regions including the genu of the CC, the right posterior limb of internal capsule, and the right splenium of the CC. From NODDI, PAT showed increased ODI in the LSLF and increased FISO in the right splenium of the CC. FICVF showed a significant decrease in the LSLF while increased in the left anterior corona radiata and the CC. Furthermore, the average values of MD and FICVF were significantly correlated with DS and AS. Hence the results of this study suggest that there are regional changes in WM in DE patients which may contribute to further understanding of the pathological mechanism of DE.

## INTRODUCTION

The tear film is an optical refractive interface covering the outermost ocular surface and is composed of lipids, mucins and electrolytes [1]. Dry eye (DE) is a multi-factorial disease in which the tear film loses stability. Tear film instability leads to increased aberrations and scattering in higher-order aberrations, thus reducing visual quality [2]. In addition, patients with DE may experience pain, burning, itching, dryness [3], persistent tear deficiency and a combination of long-term chronic inflammation, peripheral nerve injury and ocular inflammation, presenting as chronic neuropathic pain [4]. However, symptoms of DE may be severe and unaccompanied by equivalent signs.

The mechanism of DE disease has not been fully explained. Chronic inflammation has been viewed as its central mechanism but a more recent definition [5, 6] suggests that it is caused by inflammation induced by the hypertonic tear film, neurogenic stress response, and neurosensory abnormalities. These reactions further promote inflammatory damage to the ocular surface and glands, affect the generation of blood vessels and lymphatics, and aggravate inflammatory reactions, leading to a vicious cycle of inflammation. As 2017 International Dry Eye Workshop II (DEWS II) defined, neurosensory abnormalities” was included in the definition of DE for the first time [7]. A growing number of studies suggest that neuropathic symptoms are associated emotional disorders in dry eye. Nervous anxiety is a normal reaction to stimuli such as inflammation, and neurological symptoms can result from somatic neuropathy or somatosensory irritation of the cornea or conjunctiva. Sympathetic and parasympathetic nerve endings secrete neurotransmitters which participate in the maintenance of ocular surface homeostasis and regulate tear secretion. Corneal nerve fibers originate from the trigeminal nerve, the sensory nucleus of which receives afferent nerve signals from the cornea or conjunctiva. These signals are transmitted to the salivary nucleus on the brain stem, exciting the parasympathetic nerve that innervates the lacrimal gland, and stimulating it to secrete the aqueous component of the tears [8]. So abnormal neuroregulation can lead to abnormal tear secretion.

Diffusion tensor imaging (DTI) is a new functional magnetic resonance imaging (fMRI)-based technology which has the advantage of capturing images of white matter, and is the most commonly used non-invasive method to study brain tissue. DTI has been widely used to investigate a range of mental or neural illness, such as schizophrenia [9], Alzheimer’s disease [10], multiple sclerosis [11] and depression [12]. Many studies have shown that neuropsychiatric diseases are accompanied by white matter abnormality, providing insights into the pathogenesis and pathological mechanism of the disease. NODDI is an imaging method which allows evaluation of nerve axon and dendrite structure. Compared with DTI, NODDI may more accurately reflect water molecule diffusion within the tissue microenvironment and may therefore better reflect brain development and a variety of neurological diseases.

Our previous studies [13–15] found that some ophthalmic diseases can cause brain regional changes. Using fMRI, researchers found that DE may lead to dysfunction of specific brain regions [16]. Similarly, studies have suggested that abnormal regional homogeneity of the limb-cortical circuit in patients with DE, and this dysfunction may be related to the pathological mechanism of DE [17]. Abnormality of this kind may explain cognitive impairment, psychiatric symptoms and depressive mood in patients with DE. White matter nerve fiber bundles are fundamental to signal transmission in many chronic diseases and are damaged in brain disorders. No research has investigated whether white matter nerve fiber bundles are damaged in DE, so this study aims to determine whether white matter is abnormal in DE patients, using DTI and NODDI, and to increase understanding of the pathophysiology of DE.

## METHODS

### Participants

#### Subjects

The subjects from the First Affiliated Hospital of Nanchang University will be divided into two groups. Diagnosis of DE based on the expert consensus on clinical diagnosis and treatment of dry eye [18], Participants were selected as a patient group (PAT) if they had DE symptoms (OSDI ≥ 13) and at least one eye meeting the following criteria: Non-Invasive Keratograph Break-Up Time (NIKBUT) <10 s; tear osmolarity ≥308 mOsm/L; corneal fluorescein staining  >5 spots; conjunctival lissamine green staining >9 spots; lid margin staining  ≥2 mm length and/or ≥25% sagittal width, and healthy subjects were included in a control group (HC). Age (years), sex, weight (kg), handedness, duration of DE (months), and monocular best-corrected visual acuity were recorded. This study was conducted with the approval of the Hospital ethics committee, and declarations of informed consent were signed by all participants.

#### Inclusion criteria

(1) Presence of at least two of the following symptoms: burning, itching, foreign body sensation, blurred vision, photophobia; (2) Age between 20 and 65; (3) No local or systemic use of any drugs in the preceding 2 weeks.

#### Exclusion criteria

(1) Diagnosis of rheumatoid arthritis, Sjogren’s syndrome or other systemic immune diseases; (2) Local or systemic glucocorticoids, immunosuppressants and other drugs used within 1 week preceding treatment; (3) History of ophthalmic surgery; (4) History of neurological diseases and other serious systemic diseases; (5) History of conjunctival, corneal or iris disease.

### MRI data acquisition

#### Image processing

##### Data preprocessing

FMRIB (FSL) (http://www.fmrib.ox.ac.uk/fsl) was used to preprocess MRI data. After the original DICOM data were transferred into NIfTI format using MRIcron software, the DTI data were vortex-corrected by FMRIB to align the data to the B = 0 image. Magnetic field heterogeneity, head movement artifacts and scalp and skull images were removed. The Matlab NODDI toolbox (UCL, UK) (http://nitrc.org/projects/noddi_tolbox) was used for computation and fitting of the NODDI microstructure model. The NODDI index obtained by fitting includes isotropic volume fraction (FISO), intra-cellular volume fraction (FICVF), and orientation dispersion index (ODI).The FSL toolkit FDT3.0 (http://fsl.fmrib.ox.ac.uk/fsl/fslwiki/FTD) was applied to generate various diffusion tensor parameters and obtain values of fractional anisotropy (FA), axial diffusivity (AD) and radial diffusivity (RD).

##### Data analysis

The difference between the DE group and HC group was calculated using the tract-based spatial statistics (TBSS) method, and the FA data were preprocessed. The FA graphs of data from subjects in the two groups were registered to the standard FMRIB template and converted to the standard Montreal Neurological Institute 152(MNI152) space (1 × 1 × 1 mm^3^). The FA skeleton was generated in the standard space, reflecting the distribution of the main white matter fiber bundles in each group. The average FA images of all participants were projected onto FMRIB58_FA to obtain the average FA skeleton. The specific fiber bundles were determined using The Johns Hopkins University (JHU) standard.

Based on the threshold-free clustering enhancement (TFCE) statistical image, the voxel random arrangement comparison test of HC and PAT (5000 permutations) was carried out in FSL. The statistical threshold value *p* < 0.05 was set in all statistical graphs, and the family error was corrected ( FWE).Subsequently, the other DTI and NODDI values of this study, namely AD, RD, FISO, FICVF and ODI values, were analyzed in the same way as FA between groups.

### The questionnaire

The hospital anxiety and depression scale (HADS) measures symptoms of anxiety and depression by asking patients about their symptoms in the previous week. HADS consists of the anxiety subscale measuring anxiety and the depression subscale measuring depressive symptoms. Each subscale has 7 items with a score range of 0 to 21, and each item has 4 options with a score of 0 to 3. Criteria: 0–7 normal, 8–11 possible presence of anxiety and depression, ≥11 mood disorders may exist [19].

### Statistical analysis

This study used SPSS 25.0 statistical analysis software. The independent samples *T*-test was used to compare the demographic and clinical parameters of two groups and *p*-values < 0.05 were considered statistically significant. Receiver operating characteristic curve (ROC) curve analysis was used to distinguish the DTI and NODDI values of different white matter areas of the brain in PAT, and the area under the curve (AUC), sensitivity and specificity were obtained. Pearson’s correlation was used to assess the relationship between DTI and NODDI values and AS and DS values.

### Ethical approval and consent to participate

The study methods and protocols were approved by the Medical Ethics Committee of the First Affiliated Hospital of Nanchang University (Nanchang, China) and followed the principles of the Declaration of Helsinki. All subjects were notified of the objectives and content of the study and latent risks, and then provided written informed consent to participate.

### Availability of data and materials

The datasets used and/or analyzed during the present study are available from the corresponding author on reasonable request.

## RESULTS

### Demographic information

No significant differences were found between the groups in body weight (*p* = 0.853) or age (*p* = 0.873), while differences were found in monocular BCVA (right *p* = 0.122; left *p* = 0.163). Both groups were right-handed. Details are presented in Table 1.

**Table 1:** Basic information of participants in the study.

**Condition**	**DE**	**HCs**	***t***	*P*-value^*^
Male/Female	7/18	7/18	N/A	>0.99
Age (years)	55.68 ± 8.73	56.29 ± 6.82	0.272	0.873
Weight (kg)	56.92 ± 8.59	57.19 ± 9.16	0.216	0.853
Handedness	25R	25R	N/A	>0.99
Duration of DE (mons)	10.72 ± 3.86	N/A	N/A	N/A
Best-corrected VA-left eye	0.94 ± 0.36	1.12 ± 0.19	−0.915	0.163
Best-corrected VA-right eye	0.91 ± 0.43	1.09 ± 0.22	−0.987	0.122

Independent *t*-tests comparing two groups (*p* < 0.05). Abbreviations: HCs: healthy controls; N/A: not applicable; DE: dry eye; VA: visual acuity.

### DTI differences

**Figure 1:**
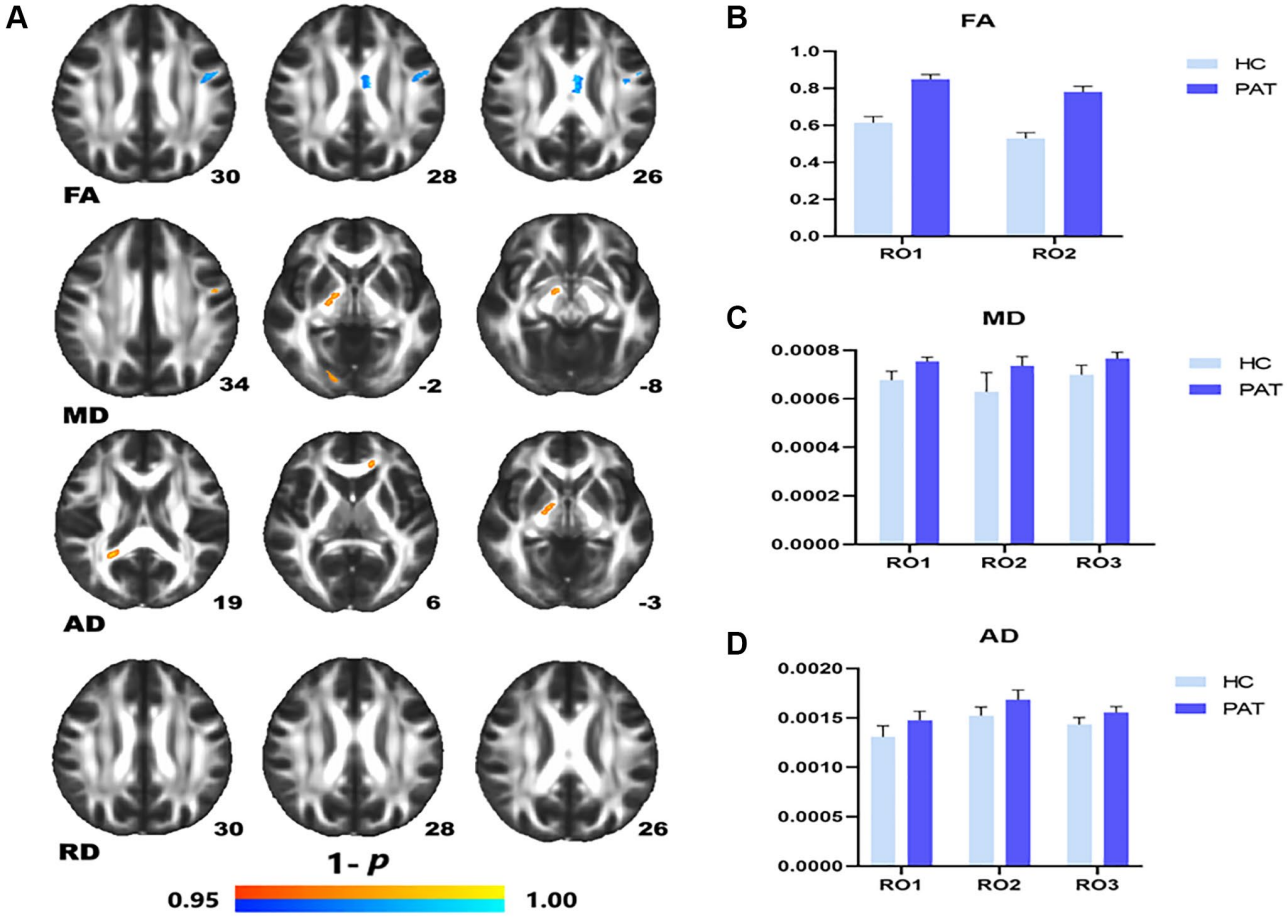
**Comparison of DTI value of HC and PAT group.** Mean DTI values between PAT and HC groups (**A**–**C**). (**A**) ROI1-left superior longitudinal fasciculus; ROI2-body of corpus callosum. (**B**) ROI1-left superior longitudinal fasciculus; ROI2-right posterior limb of internal capsule. ROI3-right posterior thalamic radiation. (**C**) ROI1-genu of corpus callosum; ROI2-right posterior limb of internal capsule; ROI3-right splenium of corpus callosum. Significant difference in mean DTI value between HC and PAT group. (**D**) The red area indicates that the value of PAT is higher than HC and blue areas indicate lower values. Abbreviations: FA: fractional anisotropy; MD: mean diffusivity; AD: axial diffusivity; HC: healthy controls; PAT: patient; ROI: region of interest.

Compared with the HC, the FA value of PAT was significantly lower in the left superior longitudinal fasciculus (LSLF) and corpus callosum (CC) (Figure 1A (blue) and Table 2). Higher DTI values in the brain areas were the LSLF (MD), right posterior limb of the internal capsule (MD), the right posterior thalamic radiation (MD), the genu of CC (AD), right posterior limb of internal capsule (AD) and right splenium of the CC (AD) (Figure 1A (red) and Table 2). The ROI signal values of the two groups were shown in Figure 1B–1D.

**Table 2:** Significant differences of DTI values between different brain WM regions of PAT and HC group.

**A PAT>HC**								
**Parameter**	**WM regions (PAT vs. HC)**	**MNI coordinates**			**Voxels number**	***T* value**	***P* value**	**Cluster**
		**X**	**Y**	**Z**				
**MD**	Left Superior longitudinal fasciculus	125	121	100	117	7.61	0.00008	ROI1
	Right Posterior limb of internal capsule	80	121	68	125	5.31	0.003	ROI2
	Right Posterior thalamic radiation	59	57	79	94	4.94	0.006	ROI3
**AD**	Genu of corpus callosum	103	160	79	109	6.37	0.0002	ROI1
	Right Posterior limb of internal capsule	74	120	74	192	5.36	0.002	ROI2
	Right Splenium of corpus callosum	67	76	90	106	5.47	0.001	ROI3
**B PAT<HC**								
**Parameter**	**WM regions (PAT vs. HC)**	**MNI coordinates**			**Voxels number**	***T* value**	***P* value**	**Cluster**
		**X**	**Y**	**Z**				
**FA**	Left Superior longitudinal fasciculus	129	116	101	185	−6.78	0.0001	ROI1
	Body of corpus callosum	97	116	101	132	−6.25	0.0003	ROI2

Between-group differences in DTI parameters. Abbreviations: FA: fractional anisotropy; MD: mean diffusivity; AD: axial diffusivity; HC: Healthy controls; PAT: patient; N/A: not applicable.

### NODDI differences

**Figure 2:**
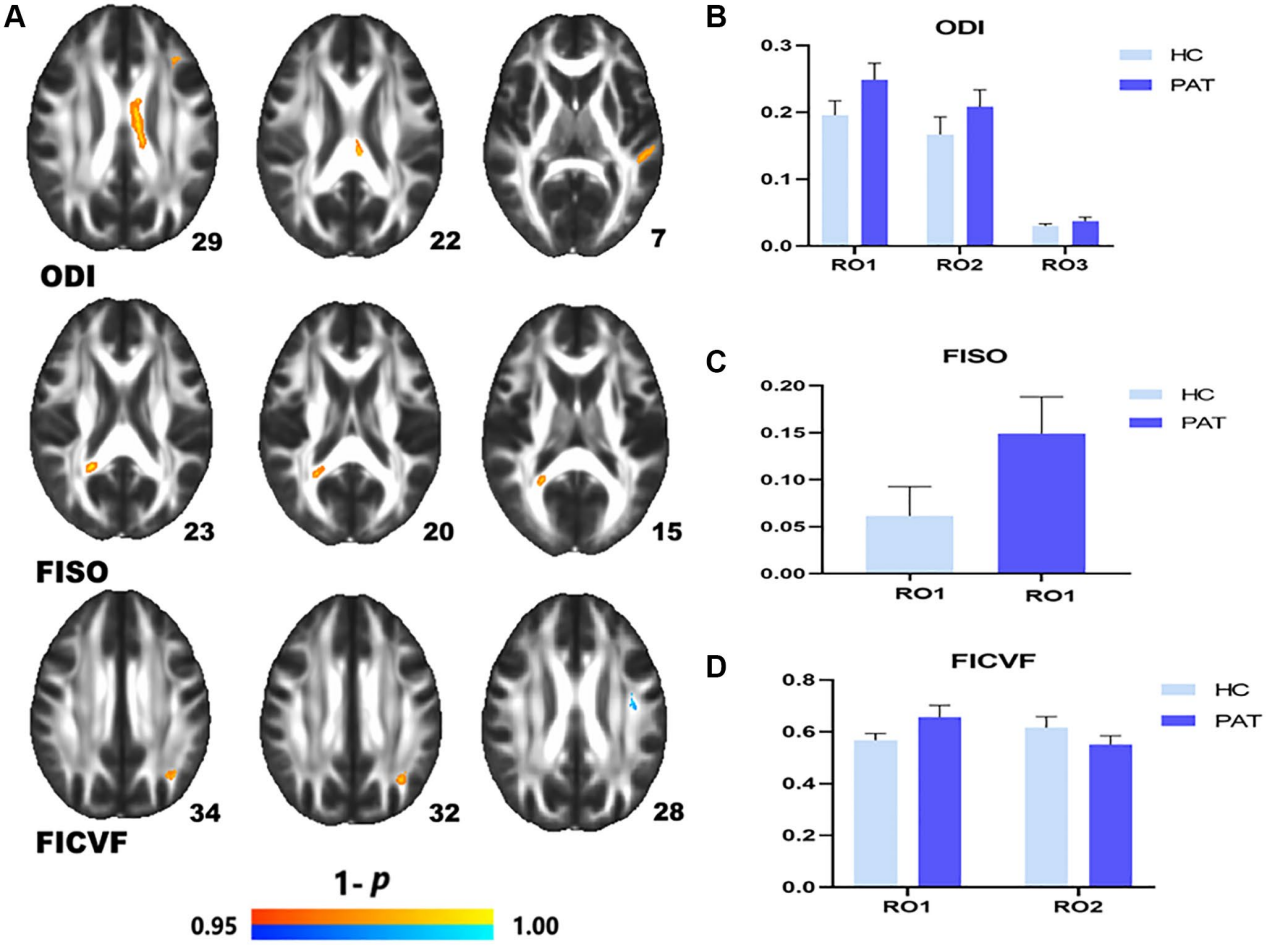
**Comparison of NODDI value of HC and PAT group.** Mean NODDI values between PAT and HC groups (**A**–**C**). (**A**) ROI1-left anterior corona radiata; ROI2-body of corpus callosum; ROI3-left superior longitudinal fasciculus. (**B**) Right splenium of corpus callosum. (**C**) ROI1-left posterior corona radiata; ROI2-left superior longitudinal fasciculus. Significant difference in mean NODDI value between HC and PAT group (**D**) The red area indicates that the value of PAT is higher than HC and blue areas indicate lower values. Abbreviations: ODI: orientation dispersion index; FISO: isotropic volume fraction; FICVF: intra-cellular volume fraction; HC: healthy controls; PAT: patient; ROI: region of interest.

Compared with the HCs, DE patients had significantly higher NODDI values in the left anterior corona radiata (ODI), the CC (ODI), LSLF (ODI), the right splenium of CC (FISO), the left posterior corona radiata (FICVF) (Figure 2A (red) and Table 3). Lower NODDI values in the brain areas were LSLF (FICVF) (Figure 2A (blue) and Table 3). The ROI signal values of the two groups were shown in Figure 2B–2D.

**Table 3:** Significant differences of NODDI values between different brain WM regions of PAT and HC group.

**A PAT>HC**								
**Parameter**	**WM regions (PAT vs. HC)**	**MNI coordinates**			**Voxels number**	***T* value**	***P* value**	**Cluster**
		**X**	**Y**	**Z**				
**ODI**	Left Anterior corona radiata	115	149	91	93	5.17	0.002	ROI1
	Body of corpus callosum	99	107	99	216	4.18	0.011	ROI2
	Left Superior longitudinal fasciculus	132	87	75	87	4.54	0.007	ROI3
**FISO**	Right Splenium of corpus callosum	67	76	90	132	6.86	0.0005	ROI1
**FICVF**	Left Posterior corona radiata	118	67	92	83	5.03	0.003	ROI1
**B PAT < HC**								
**Parameter**	**WM regions (PAT vs. HC)**	**MNI coordinates**			**Voxels number**	***T* value**	***P* value**	**Cluster**
		**X**	**Y**	**Z**				
**FICVF**	Left Superior longitudinal fasciculus	118	67	92	79	−4.56	0.007	ROI2

Between-group differences in NODDI parameters. Abbreviations: ODI: orientation dispersion index; FISO: isotropic volume fraction; FICVF: intra-cellular volume fraction; HC: Healthy controls; PAT: patient; N/A: not applicable.

### Receiver operating characteristic curve

**Figure 3:**
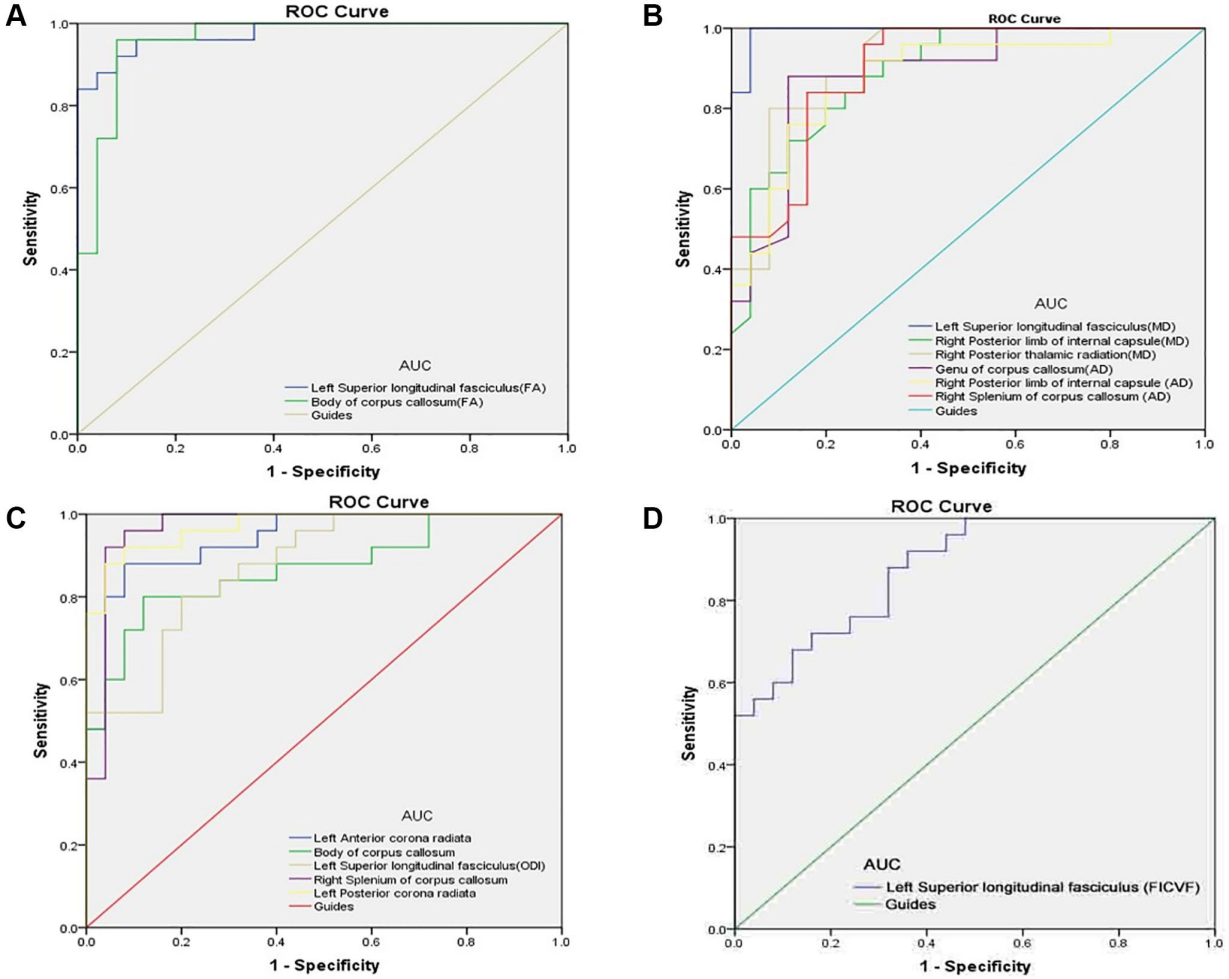
**ROC curve analysis of the mean DTI and NODDI values for altered brain regions.** (**A**) The area under the ROC curve were 0.976, (*p* < 0.0001; 95% CI: 0.941–1.000) for left superior longitudinal fasciculus (FA); body of corpus callosum (FA) 0.960, (*p* < 0.0001; 95% CI: 0.908–1.000). (**B**) The area under the ROC curve were 0.994 (*p* < 0.0001; 95% CI: 0.979–1.000) for left superior longitudinal fasciculus (MD); right posterior limb of internal capsule (MD) 0.891, (*p* < 0.0001; 95% CI: 0.804–0.979); right posterior thalamic radiation (MD) 0.918, (*p* < 0.0001; 95% CI: 0.841–0.994); genu of corpus callosum (AD) 0.888, (*p* < 0.0001; 95% CI: 0.794–0.982); right posterior limb of internal capsule (AD) 0.880, (*p* < 0.0001; 95% CI: 0.784–0.976); right splenium of corpus callosum (AD) 0.900, (*p* < 0.0001; 95% CI: 0.816–0.984). (**C**) The area under the ROC curve were 0.941, (*p* < 0.0001; 95% CI: 0.879–1.000) for left anterior corona radiata; body of corpus callosum 0.867, (*p* < 0.0001; 95% CI: 0.765–0.969); left superior longitudinal fasciculus (ODI) 0.874, (*p* < 0.0001; 95% CI: 0.780–0.967); right splenium of corpus callosum 0.968, (*p* < 0.0001; 95% CI: 0.916–1.000); left posterior corona radiata 0.971, (*p* < 0.0001; 95% CI: 0.934–1.000). (**D**) The area under the ROC curve were 0.880 (*p* < 0.0001; 95% CI: 0.790–0.970) for left superior longitudinal fasciculus (FICVF). Abbreviations: FA: fractional anisotropy; MD: mean diffusivity; AD: axial diffusivity; RD: radial diffusivity; ODI: orientation dispersion index; FISO: isotropic volume fraction; FICVF: intra-cellular volume fraction; AUC: area under the curve; ROC: receiver operating characteristic.

The ROC curve was used to evaluate the value of DTI and NODDI parameters in the two groups for diagnostic. AUC was positively correlated with diagnostic significance. The results showed that the value of DTI and NODDI in different regions of brain were significantly different in the two groups. Hence, as markers, DTI and NODDI values are useful for distinguishing DE from HC. Results of AUC were as follows: DTI: The AUC were 0.976 for LSLF (FA); 0.960 for CC (FA) (Figure 3A, PAT < HC); 0.994 for LSLF (MD); 0.891 for right posterior limb of internal capsule (MD); 0.918 for right posterior thalamic radiation (MD); 0.888 for genu of CC (AD); 0.880 for right posterior limb of internal capsule (AD); and 0.900 for right splenium of CC (AD) (Figure 3B, PAT > HC). NODDI: The AUC was 0.941 for the left anterior corona radiata; 0.867 for CC; 0.874 for the LSLF (ODI); 0.968 for the right splenium of CC; 0.971 for the left posterior corona radiata (Figure 3C, PAT > HC); and 0.880 for the LSLF (FICVF) (Figure 3D, PAT < HC). All *p* values were < 0.0001 and AUC were over 0.867.

### Correlation analysis

**Figure 4:**
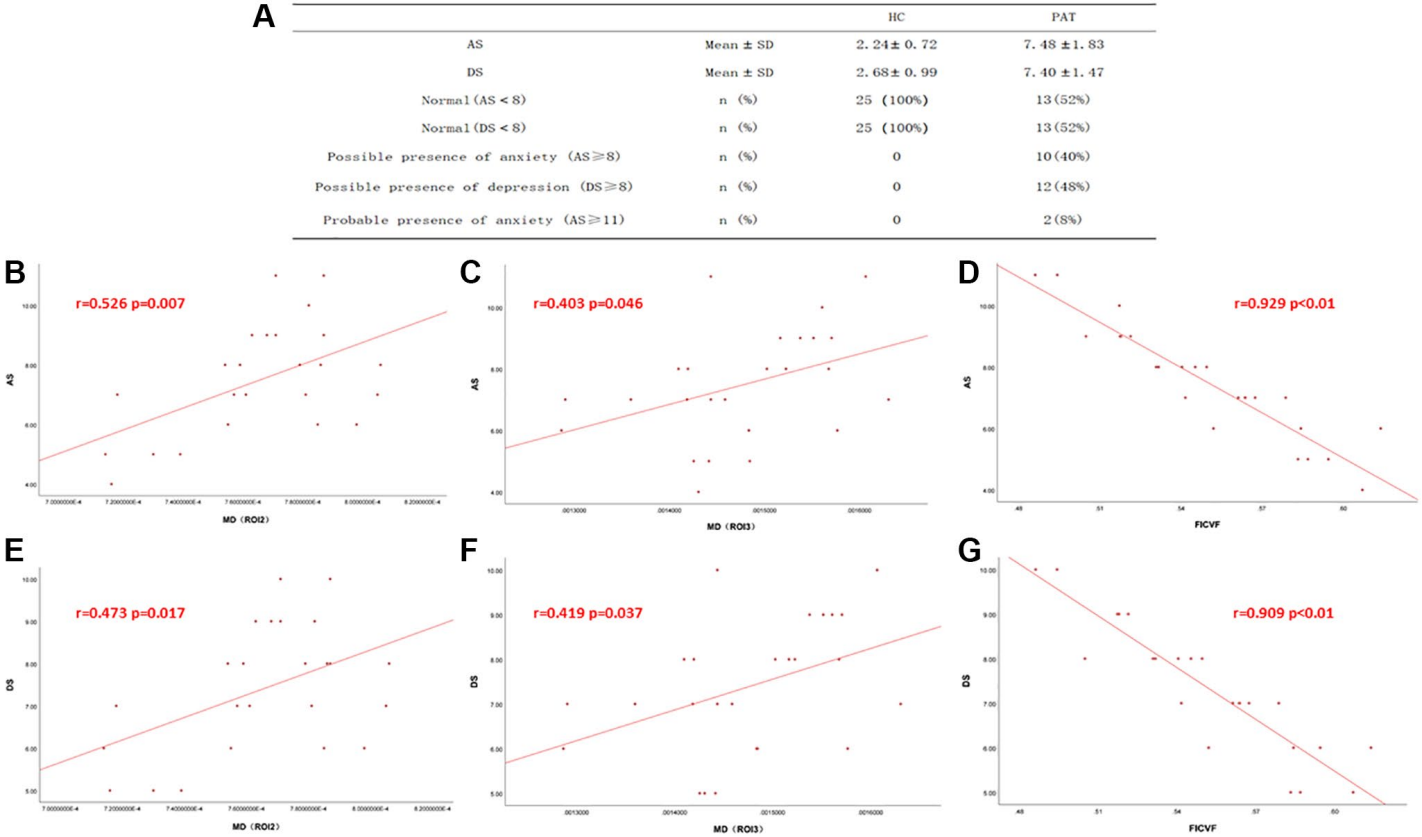
**Correlations between the DTI values, NODDI values and AS, DS.** (**A**) The value of AS and DS in two groups. (**B**–**G**) In There was a positive correlation with the values of MD and DS, AS. And the mean value of FICVF was negatively correlated with DS and AS. Abbreviations: DS: depression score; AS: anxiety score; MD: mean diffusivity; FICVF: intra-cellular volume fraction; HC: Healthy controls; PAT: patient.

AS and DS values of HC group and PAT group were shown in Figure 4A. In the HC group, AS and DS were not significantly correlated with DTI and NODDI values. In the DE group, the value of FICVF was negatively correlated with DS (r = −0.909, *p* < 0.01) (Figure 4G) and AS (r = −0.929, *p* < 0.01) (Figure 4D) While the MD (ROI2) value was positively correlated with DS (r = 0.473, *p* = 0.017) (Figure 4E) and AS (r = 0.526, *p* = 0.007) (Figure 4B). The mean value of MD (ROI3) was positively correlated with the DS (r = 0.419, *p* = 0.037) (Figure 4F) and AS (r = 0.403, *p* = 0.046) (Figure 4C).

## DISCUSSION

Dry eye is one of the most common eye diseases, accounting for 5% to 50% of dry eye cases worldwide [20], seriously affecting patients’ work and life and even causing social-psychological problems such as depression [21], stress [22], anxiety and sleep disorders [23]. However, the multifactorial cycle and pathological mechanism of dry eye has not been fully clarified. The results of this study suggest that there are microstructural changes in WM in DE patients, which may be the basis of mood abnormalities in DE patients.

### DTI and NODDI characteristics

DTI is a very useful imaging technique in the clinical diagnosis and study of the microstructure of neural tissues, helping us to better understand the neurophysiological mechanisms of many diseases. DTI can quantitatively analyze the diffusion of water molecules in three-dimensional space within a brain region of interest, thus revealing the structure of WM fiber bundles. The MD in DTI reflects the range of diffusion motion of water molecules per unit time. The increase of MD and AD values may be partly caused by demyelination [24], although other factors such as axon loss, fiber density, axon diameter, and cell membrane integrity may also be responsible. Animal experiments have shown that demyelination after axonal injury often leads to an increase in transverse (short-axis) diffusivity and a decrease in axial (long-axis) diffusivity [25].

FA is very sensitive to white matter changes, reflecting the integrity of myelin sheath, axon density and any changes in diameter [26]. Significant FA decreases in the LSLF and the CC may reflect reduced neural integrity within these regions in DE, due to demyelination or axonal injury [27]. NODDI is an emerging method based on magnetic resonance diffusion imaging [28], which compared with DTI offers more complete and accurate indication of water diffusion behavior in complex brain tissues. ODI is a measure of changes in the direction of nerve processes. In our study, significantly raised ODI values perhaps suggesting a loss of fiber coherence and relatively maintained neuronal fiber density [29, 30].

### Application of DTI and NODDI

**Figure 5:**
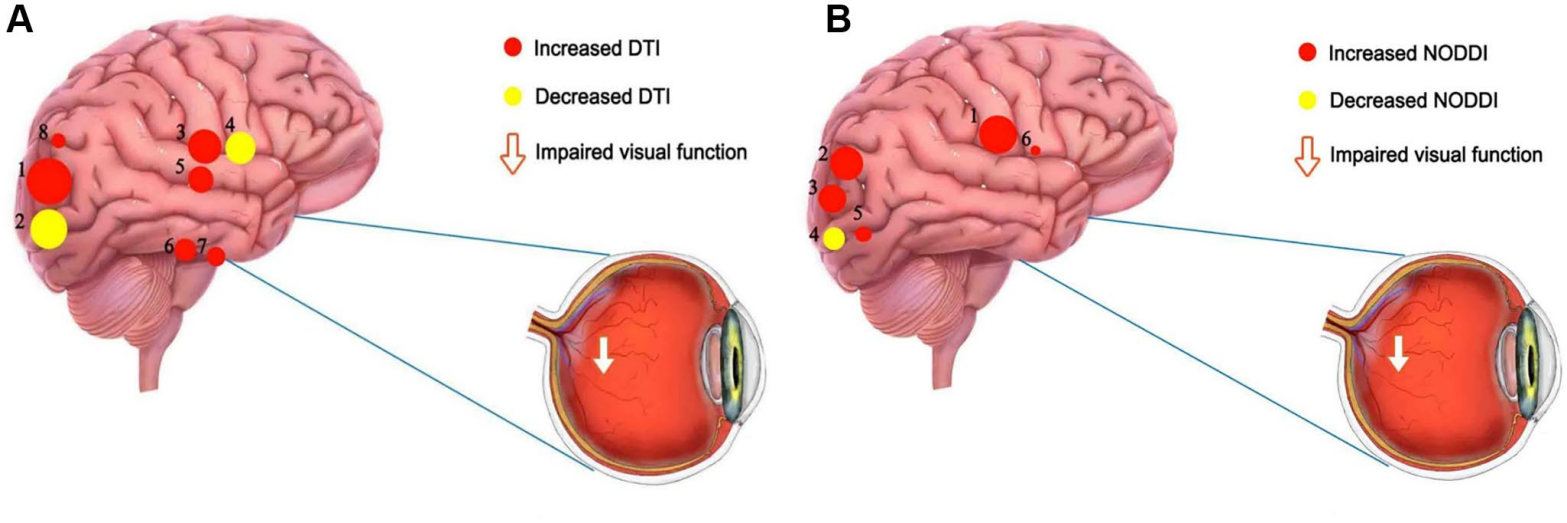
**The mean DTI and NODOI values of altered brain regions.** (**A**) Compared with the HCs, the DTI values of the following regions were decreased to various extents: 2- left superior longitudinal fasciculus (FA) (t = −6.78), 4- body of corpus callosum (FA) (t = −6.25). Compared with the HCs, the DTI values of the following regions were increased to various extents: 1- left superior longitudinal fasciculus (MD) (t =7.61), 7- right posterior limb of internal capsule (MD) (t = 5.31), 8- right posterior thalamic radiation (MD) (t = 4.94), 3- genu of corpus callosum (AD) (t = 6.37), 6- right posterior limb of internal capsule (AD) (t = 5.36), 5- right splenium of corpus callosum (AD) (t = 5.47). (**B**) Compared with the HCs, the NODOI values of the following regions were increased to various extents: 2- left anterior corona radiata (t = 5.17), 6- body of corpus callosum (t = 4.18), 5- left superior longitudinal fasciculus (ODI) (t = 4.54), 1- right splenium of corpus callosum (t = 6.86), 3- left posterior corona radiata (t = 5.03). Compared with the HCs, the NODOI values of the following regions were decreased to various extents: 4- left superior longitudinal fasciculus (FICVF) (t = −4.56). Abbreviations: HCs: healthy controls; DTI: diffusion tensor imaging; FA: fractional anisotropy; MD: mean diffusivity; AD: axial diffusivity; ODI: orientation dispersion index; FISO: isotropic volume fraction; FICVF: intracellular volume fraction.

Previous studies have applied DTI and NODDI in ophthalmology (Table 4), but to our knowledge, the present study is the first to assess WM changes in patients with dry eye using DTI combined with NODDI. Our study found that compared with the HCs, DTI and NODDI values were reduced in some brain regions (Figure 5). ROC results were used to verify DTI and NODDI analysis, and showed areas under the ROC curves greater than 0.7, indicating reliable accuracy. Therefore, it can be concluded that regional brain WM is altered in DE patients compared with HC. In addition, DTI and NODDI values reflect regional changes in white matter, and abnormalities in WM may be the basis for pain and mood disorders in DE patients (Table 5).

**Table 4:** DTI and NODDI applied in ophthalmologic disease.

	**Author**	**Year**	**Disease**
**DTI**	Liu et al. [31]	2020	Monocular blindness
	Tian et al. [32]	2019	Neuromyelitis Optic Neuritis
	Wang et al. [33]	2018	Primary Open-Angle Glaucoma
	Lee et al. [34]	2018	Dysthyroid Optic Neuropathy
	Zhong et al. [35]	2017	Monocular blindness
	Gupta et al. [36]	2016	Strabismic amblyopes
	Huang Xin et al. [37]	2016	Comitant strabismus
	Schoemann et al. [38]	2014	Primary open-angle glaucoma
	Schmidt et al. [39]	2014	Primary open-angle glaucoma
	Michelson et al. [40]	2013	Glaucoma
**NODDI**	Kang et al. [41]	2021	Anterior Visual Pathway Compression

Abbreviations: DTI: diffusion tensor imaging; NODDI: neurite orientation dispersion and density imaging.

**Table 5:** Alternation of brain white matter regions and its potential impact.

**Brain regions**	**Experimental results**	**Brain function**	**Anticipated results**
Thalamic	PAT>HC (MD)	transmit, process and respond visual information and pain information	Visual impairment and persistent pain symptoms
Left Superior longitudinal fasciculus	PAT < HC (FA, FICVF) PAT>HC (MD, ODI)	associated with affective disorders	Depression and anxiety
Right Posterior limb of internal capsule	PAT>HC (MD, AD)	associated with different aspects of emotion, motivation, cognition processing, and decision-making	Social and emotional problems
Corpus callosum (Body, Splenium, Genu)	PAT < HC (FA) PAT>HC (AD, ODI, FISO)	integrate motor, emotional, and cognitive functions; part of the default model network	Social and emotional problems
Corona radiata	PAT>HC (ODI, FICVF)	attentional control	Cognitive impairment

Abbreviations: HC: healthy controls; PAT: patient controls.

### Relationship between brain regions, DE and HADS

The thalamus is located around the bilateral third ventricle and is the largest oval mixed nucleus of gray matter in the diencephalon. The thalamus has specific connections with the corresponding cerebral cortex. As a completely functional network, the cerebellum-thalamus can transmit, process and respond to visual and pain information within and beyond the visual brain [42, 43]. In this study, MD values of the right posterior thalamus were significantly different between DE and HC. Pan et al. [44] used the global brain functional connectivity (GFC) method at voxel level to study the differences in functional tissue between DE patients and healthy controls. The GFC changes in the cerebellum-thalamic-cortex network in DE patients may be related to visual impairment and persistent pain symptoms, and reduced GFC may lead to a continuous stress response and reduced pain threshold. The superior longitudinal fasciculus (which contains bidirectional fibers running from the frontal to the parietal, temporal and occipital lobes) is a communication link, connecting the “control network” region of the frontal parietal lobe with other regions. Studies have shown that the FA value of LSLF is decreased in patients with major depression [45, 46], and in affective disorders [47, 48].

The CC is the largest WM fiber bundle, connecting the bilateral cerebral hemispheres. The fibers in the knee of the corpus callosum are mainly connected with the temporal and parietal lobes. Abnormality of WM in schizophrenia or psychosis most commonly occurs in the CC [49]. In the present study, the FA values of the LSLF and CC were decreased in DE patients, while ODI and FISO values were increased, and these findings may be related to the affective disorders caused by DE. The inner capsule is a bundle of fibers that perform primary motor functions. Poulakis [50] found that the knee of the CC, the forelimb of the inner capsule, and the integrity of the coronary fiber bundle after radiation may be used to assess individuals’ risk of future cognitive decline. In addition, the increase in MD and AD values of the right posterior limb of the internal capsule may be related to the social and emotional problems of DE.

**Figure 6:**
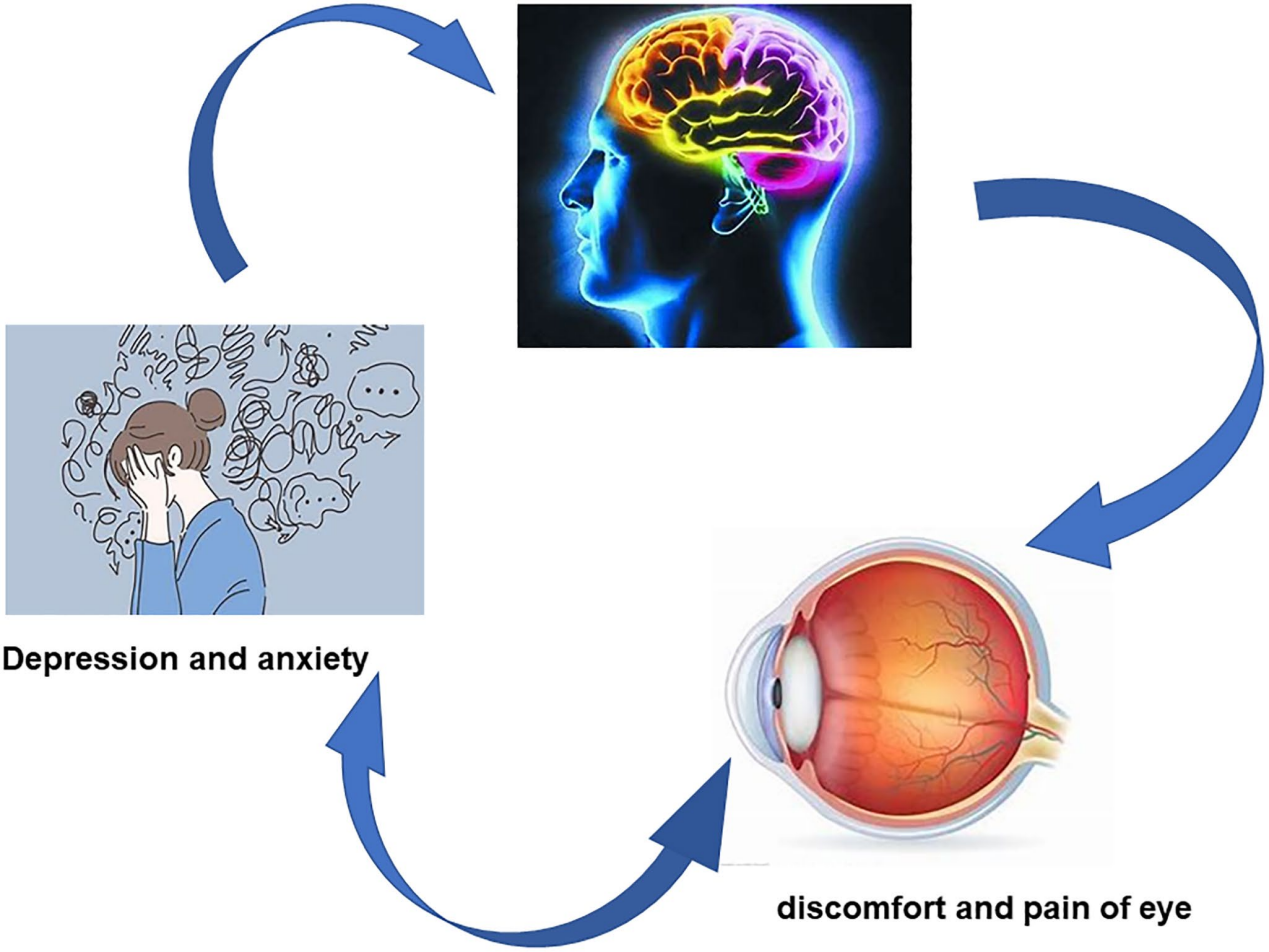
**Correlation between DTI and NODDI values and emotional disorder.** Compared with HC group, the values of DTI and NODDI in some brain white matter regions were significantly different in PAT group, and patients with dry eye were more prone to depression and anxiety. Abbreviations: HC: healthy controls; PAT: patient controls.

HADS is an effective method to evaluate depression and anxiety in patients with DE. Patients with severe symptoms requiring psychological treatment were not included in our study, and HADS in patients with DE was higher than that in the normal group, reflecting the correlation between dry eye and HADS. Persistent eye discomfort and visual impairment caused by DE can lead to anxiety, depression and other mood disorders. Research showed in rats that depression can lead to increased secretion of IL-1B and TNFα and reduced tear secretion [51]. Depressive symptoms are associated with subjective dry eye symptoms, but not with more objective measures (tear break-up time, Schirmer test and corneal staining) [52]. Correlations between mean values MD and FICVF and both AS and DS suggest that the regional changes of WM in DE patients may be related to the patient’s anxiety and depression (Figure 6).

### Limitations

This study has some limitations: 1) DTI adopts rapid prototyping technology, is very sensitive to the movement of small molecules, and artifacts caused by physiological movement may affect its results; 2) The small samples limits the credibility of the conclusions, and future studies with larger samples are needed to verify the method and results.

## CONCLUSIONS

In conclusion, regional changes in brain WM in DE patients may help reveal the pathological mechanism of dry eye and provide a new evaluation index for clinical diagnosis. It is hoped that the current research findings will draw attention to the psychological state of patients with dry eye and lead to further research and treatment for DE patients.
