# Correction for: Altered regional brain white matter in dry eye patients: a brain imaging study

**DOI:** 10.18632/aging.204090

**Published:** 2022-05-15

**Authors:** Yun-Qing Luo, Rong-Bin Liang, San-Hua Xu, Yi-Cong Pan, Qiu-Yu Li, Hui-Ye Shu, Min Kang, Pin Yin, Li-Juan Zhang, Yi Shao

**Affiliations:** 1 Department of Ophthalmology, The Second Affiliated Hospital of Nanchang University, Jiangxi Province Ocular Disease Clinical Research Center, Nanchang, Jiangxi, PR 330006, China; 2 Department of Ophthalmology, The First Affiliated Hospital of Nanchang University, Jiangxi Province Medical Imaging Research Institute, Nanchang, Jiangxi, PR 330006, China

**This article has been corrected:** The authors changed the order of the affiliations 1 and 2 and added new affiliation to the first author due to the job transfer. The new affiliations are presented below.

Yun-Qing Luo^1,2,*^, Rong-Bin Liang^1,*^, San-Hua Xu^1,*^, Yi-Cong Pan^1^, Qiu-Yu Li^1^, Hui-Ye Shu^1^, Min Kang^1^, Pin Yin^1^, Li-Juan Zhang^1^, Yi Shao^1^

^1^Department of Ophthalmology, The First Affiliated Hospital of Nanchang University, Jiangxi Province Medical Imaging Research Institute, Nanchang 330006, Jiangxi, PR China

^2^Department of Ophthalmology, The Second Affiliated Hospital of Nanchang University, Jiangxi Province Ocular Disease Clinical Research Center, Nanchang 330006, Jiangxi, PR China

^*^Equal contribution

